# Role of politically motivated internet addiction and ideological passion in linking college student's mental health education and wellbeing

**DOI:** 10.3389/fpsyt.2022.973520

**Published:** 2022-08-12

**Authors:** Shuyu Meng

**Affiliations:** ^1^Admissions and Employment Office, Xinyang Normal University, Xinyang, China; ^2^School of Marxism, China University of Political Science and Law, Beijing, China

**Keywords:** ideological passion, mental health education, student's wellbeing, internet addiction, mental behavior

## Abstract

College students are increasingly reporting common mental health issues like depression and anxiety, raising severe concerns for students' psychological wellbeing. Specifically, after the emergence of Post-COVID-19, digitization caused a potential role in mitigating students' psychological concerns. Therefore, the role of mental health education has been regarded as a topic of interest in managing the issue of Chinese college students' mental wellbeing. This study intends to look into the relationship between mental health education and psychological wellbeing, along with the moderating role of politically motivated internet addiction and the ideological passion of college students. For the given reason, the random sampling method was employed for collecting data from target respondents. The study uses 750 questionnaires prepared on a five-point Likert scale that were distributed to the respondents with an expected response rate of 50%. The partial least square (PLS) software was used to analyze the data for this study. The study concludes that there is a significant moderating role of politically motivated internet addiction and ideological passion in the relationship between college students' mental health and wellbeing. The study meaningfully contributes to the body of knowledge by establishing the unique, positive moderating role of Politically motivated internet addition in strengthening the relationship which undoubtedly will assist in improving the psychological wellbeing of college students through mental health education policies and implications.

## Introduction

Since the last decade, there has been an unprecedented increase in the symptoms of stress and depression among college students (1, 2). Internationally, ~31% of college students were deemed to be suffering from mental health disorder since last year (3). It has also been observed that they suffered from scarcity and unavailability of readily available mental health educational programs. In modern times, mental health education is considered one of the fundamental factors for the better productivity and performance of students. It is essential to understand that the students are highly motivated to improve their learning performance and are provided better health-related education interventions to improve their productivity constructively (4). Likewise, rapid industrialization, digitization, and materialism have increased performance expectations from students, adding to the performance pressure (5, 6). At the same time, the students are facing the challenge of mental health for better performance and critical learning not only in mainland China but also in Taiwan and other geographical boundaries of China (7, 8). However, the student's mental health is critical for their productivity and overall academic endeavors (9). Previously, parents used to be primarily responsible for enlightening and motivating students for their prosperous psychological, academic, and professional lives (9–11). Conversely, the availability of uninterrupted internet has lately been the source of advanced-level academic and psychological aid.

Additionally, due to the emergence of informational technology, and congested professional routines, there is an apparent decline in the frequency and intensity of interaction between students and their parents. It has been replaced by controlled, managed, and efficient internet availability (5, 12, 13). Furthermore, digital mental health aides reached through mobile, and internet-based platforms, are deemed to impact individuals faced with mental health issues positively. It indicates the likely increase in internet addiction among the students; as such digital mental health therapy is considered to be easily adoptable because of lesser stigmas (14, 15). Likewise, recent studies have indicated the notable role of such digital mental health intervention in countering mental health issues (16), specifically in the case of university and college students. Likewise, the students are influenced by different motivational speakers, and they have an ideological passion for following, which has helped them fight the undue stress and psychological burden caused by faster social and academic lives (17–20). Furthermore, mental health education includes all the information, both primary and secondary, provided to students across the globe to assist them in having a check on both do and do not for an improved psychological state (21, 22). Indeed, the students with better mental health perform well in society and class activities because they have a constructive approach and do not get involved in useless activities (23–25). Additionally, ideological passion is one of the basic needs of the human personality, as human nature is dynamic, and every individual has a different set of leaves and passions (8, 24). The people who have an ideology for a better future are observed to work comparatively harder than those who lack or do not have any ideological viewpoint to follow (26, 27). The people in China are highly ideological; mainly, the student is with specific role models who help them with their motivational ideologies and passion (8, 12, 28).

On the other hand, internet addiction is increasing nowadays because people are provided with reliable internet facilities (29–31). As a result, most students across country have easy access to reliable information on their academic and personal development. Though politically directed internet addiction is not unreliable all the time, whenever it exceeds the prescribed threshold, it holds the potential to destroy college students' psychological wellbeing and careers (5, 29, 32, 33). In the same way, previous literature has found a positive relationship between ideological values and passion held by students and their ability to learn and acquire prestigious positions in their professional careers (2, 34–37). Therefore, it is necessary to pay special attention to the psychological status of college students who are exposed to the existing fast pace life and increased academic and social pressures. So, timely and appropriate mental health interventions must be applied to maintain and improve their psychological wellbeing. It remains unclear which types of interventions and elements are most effective (38). This study aimed to determine the role of politically motivated internet addiction and ideological passion as a moderator in linking college students' mental health education and wellbeing. It is critical to understand that, up to the researcher's knowledge, there is a scarcity of literature available on the above-stated variables, especially in the context of college students. Similarly, this study determines the moderating role of politically motivated internet addiction because it is increasing in educational institutes. The scope of the study is limited to improving the wellbeing of the students by linking mental health education as a critical success factor for improving and advancing the students' mental abilities and cognitive development. Therefore, further research must be conducted on how to best and innovatively use adolescent mental health education programs to enhance wellbeing and positive attitudes toward mental health.

In the same way, this study contributes to the literature by providing a robust theoretical framework developed on a different variable critical for collage students in modern times. This study is critical because it was developed to provide theoretical and practical implications for students studying in different colleges and universities to improve their wellbeing at the advanced level. It is a fact that different political associations influence students, and they utilize internet activities for such kind of propaganda (8, 12, 39, 40). In this regard, this study provides significant theoretical and practical applications that are critical to be considered by the stakeholders and the parents of students to make them work and perform in an improved manner. Though certain studies have talked in favor of positive internet-based addiction for its role in fighting the students' mental health issues, the scarcity of empirical evidence in this regard poses a significant gap. Therefore, it is believed that mental health programs and internet-based interventions need to be tested in natural settings before being formally implemented (41).

It is critical to understand that when students are provided with the opportunity to utilize the internet and their ideological passions in a productive way to improve their learning performance, their thinking abilities develop more (8, 12, 35, 42). They would perform better compared to the other students who are not provided with the opportunity to improve their mental health ability with the help of mental health education (9, 10). Therefore, there exists an identifiable gap and need across literature on finding workable strategies and interventions for developing the psychological wellbeing of the masses. Similarly, to broaden the body of knowledge on subject matter, studies need to be conducted to dig deep into the role of parents, influencers, and other agents of socialization in the development of children and adolescents' mental strength (43). In addition, the current study provides significant future directions that would be critical to consider for the expertise to contribute to the literature and deal with the unaddressed areas in the knowledge related to the wellbeing of Chinese students.

## Literature review and hypotheses development

**Figure 1:**
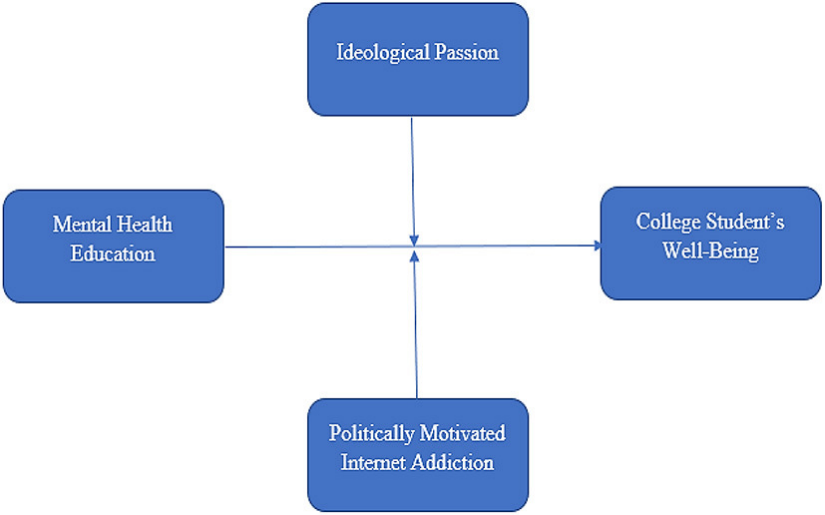
Theoretical framework.

This study's theoretical framework is developed carefully and critically, analyzing the previous literature. In this regard, the literature on mental health development and student wellbeing was analyzed to identify the significant area to contribute to the literature. In this regard, it was identified that limited studies discussed the role of mental health education in the context of students' wellbeing (44). Further, studies also recommended the importance of ideological passion for the wellbeing of the students. Conversely, studies have highlighted the adverse effects of internet addiction on the wellbeing of students (5, 8). As a result, the theoretical framework of this study was designed to contribute to the body of knowledge by providing a robust theoretical framework based on the relationship of carefully taken variables to improve the wellbeing of students. Additionally, this theoretical framework focuses on the moderating role of ideological passion and politically motivated internet addiction, considering the increased role of the internet in students' lives for making them academically and psychologically self-dependent. The theoretical framework of the research is available in Figure 1.

### Mental health education and colleges student's wellbeing

The students of any country are the primary and most valuable asset of that country because they are the future generation and have to play a critical role in the country's development. In this regard, the responsibility of the parents is to motivate their children to get an education effectively for their benefit and the country's benefit as well (12, 24). Those students who are interested in learning in their class activities are more motivated and interested in working in project-based learning (8, 45). It is because in project-based learning, the students communicate with each other, and by helping each other, they can quickly achieve their target (8, 25, 46). In comparison, such student-related engagements are mainly dependent on the level of their motivations, which is a by-product of their psychological strength and wellbeing (47). Notably, the mental health status of any student is a critical aspect of his academic life because mental health issues make it difficult for students to perform (12, 48, 49). However, different factors limit the students to enable and boost both their mental & physical health abilities. In advanced and developed countries, the students are provided with the opportunity to improve their learning ability with the help of the teacher's motivation (50). It is a fact that if the students are provided with compelling motivation by the teacher, their performance of the students would be increased to the appropriate level. Likewise, such ideological motivations can come from formal and informal mentors from one-to-one and digital sources.

Moreover, in the schools of Japan and Korea, the students are motivated to enhance their learning ability and critical thinking ability with the help of mental exercises and other physical activities that enhance both the mental and physical health of the people (24, 51). In this way, not only the mental ability of the students is being increased, but they are provided with the opportunity to get effective mental drilling and motivation by the guide to develop effective strategies to achieve their goals (9, 10, 52). The students provided with mental health education are more concerned with improving their mental health ability because they believe that to improve efficiency in their lives, their mental health ability has a critical part to play (53). It indicates that mental health-related educational and mentoring techniques play a significant role. Similarly, once motivated by internet-based aids and physical mentorship, students work harder to develop their physical and mental health abilities because they believe that with the help of effective management and measurement, their performance could be increased to the appropriate level (54, 55).

Interestingly, the students of modern times are provided with the opportunity to utilize the technology and different applications in digital smartphones to access both generic and course-related material and information. In addition, these students are effectively utilizing the technology, and their performance is appropriate because they are getting all the related information on the internet and social media to develop their cognitive and thinking abilities (56–58). It is critical to understand that students with high motivation have an increased possibility of fighting their psychological issues and ultimately performing better. On the other hand, it is a fact that the students remain at risk of time wastage due to uncontrolled and unchecked use of digital media platforms (56, 59). Like any other development across the world, the emergence of information technology is not free of negatives; if used mindlessly, it surely can hinder one's mental ability, ultimately affecting a student's academic performance (30, 60, 61). In this way, most students are wasting their time because they are not provided with the appropriate mentorship regarding social media usage. However, they are developing their perceptions based on fake news and propaganda (30, 60). In America, most students use the internet to communicate with other fellows with contradictory political mindsets, leading to unwanted arguments (9, 10). In this way, not only are the students wasting their time learning on the internet to share their political ideologies, but they are also wasting their time learning and critical thinking. Because the internet develops people's perceptions, it affects many personalities negatively (10, 62).

In contrast, students in other nations are also involved in political spates on social media, but if provided with sufficient control, check and balance, social media usage can work for their positive academic performance outcomes (9, 10, 63). It is also observed across literature that students across the UK are more inclined toward positive and meaningful internet use than US students (9, 10, 52). It is because the students of the United Kingdom believe that their self-perception must develop their efficiency and effectiveness, and they must not be motivated critically to utilize the technology in the wrong way and try to influence other people with their ideologies (9, 10, 64). In this way, the students' productivity and critical thinking ability decline because they are not provided with compelling opportunities and suggestions to improve their reception and not get involved in different kinds of politically motivated activities on the internet (30, 50). Moreover, the responsibility of the parents is to motivate the students effectively and ensure that they are not involved in any kind of activity, including the political motivation of using the internet and wasting their study time. Therefore, to cope with the issue, students need appropriate resources to improve their perception and cognitive ability to perform well (12, 27, 28, 39, 65). It is not only the responsibility of the students and the parents, but it is the responsibility of the teachers to motivate students not to use the internet in an uncontrolled manner because, with such kind of opportunity, the students will remain in a better position to improve their productivity. Additionally, the government is responsible for developing the policies effectively and ensuring that the reasonable use of internet opportunities must be provided to the students if they are involved in any kind of political activity on social media (8, 12, 45). However, the students must be provided with an opportunity on the internet to improve their learning material and thinking ability (9, 66). Resultantly, not only will the students have easy access to mentorship, but it will also help them cope with the undue stress caused by prevailing socio-economic and political concerns through a strengthened psychological state.

***H1*.*** There is a positive relationship between mental health education and college students' wellbeing*.

### Ideological passion, politically motivated internet addiction, and college student's wellbeing

The role of mental health education is to effectively educate the students to provide them with reliable information about their mental ability to improve it to the expected level (9, 10, 52). The responsibility of the school management is to ensure that the students are provided with insights on the role of mental health ability because, with the help of mental health ability and mental literacy, student performance can be increased effectively (10, 52). Significantly, students who primarily develop their mental health ability used to be the ones with extrovert personalities and an interest in keeping themselves aligned with the course material through active participation in different academic events (9, 12, 13, 52). It is critical to understand that in modern times the students are more passionate and obsessed with political affiliation because, being part of the society, they are more interested in joining any party and voting for the ideological political leader (12, 65, 67). However, by involving in such activities, the students' performance decreases because they are not provided with adequate resources to improve their performance according to the expected level. In America and Australia, the students are more politically activated because they are highly involved in political activities as they consider these little activities their future (8, 12, 30, 68). No doubt, the student's involvement in political activities is not a bad idea because, with the help of such opportunities, the students can select their careers as a politician in the future (8, 28, 69, 70). Oppositely, the students involved in such activities to create political awareness and political campaigns in society are highly motivated to utilize the services of the internet for political purposes (8, 48, 71).

It is critical to understand that the students, which have shown more interest in learning a new course, have mostly been the ones with lesser political activities, and they have been aware of the negative consequences of the wastage of time (12, 49, 72). The responsibility of the management is not only to provide the course material and the teacher to the students, but it is also the responsibility of the stakeholders to ensure that the students are not involved in different kinds of political activities because they must focus on their productivity (8, 12, 34, 39). The students who do not feel motivated by the political leaders must be provided with the opportunity to enhance their critical thinking ability and understand the critical success factor in their living standards (12, 39, 73). Therefore, it is also the responsibility of the governments and educational institutes to make students understand the difference between having political literacy and negatively engaging in politics. This way, authorities will be in a better position to let students develop an analytical understanding of the country's socio-political and economic conditions and seek necessary mentorship. However, they must focus on their productivity as a student to secure advanced degrees (8, 12, 65).

Besides, it is the responsibility of the teachers to motivate the students, as the teachers of Denmark and Norway are motivating the students not to get involved in unchecked political associations because of the excessive frequency of information available through social media (8, 12, 39, 50). In Asian countries, students are more involved in political activities, and they are highly influenced to utilize the internet for political purposes and develop the perception based on widespread online opinion (8, 12, 45). It is the extended motivation that helps the students to get rid of the traditional motivation and political players; in this way, the responsibility of the teachers and the parents is to motivate the students for their better performance and better learning (12, 49, 73, 74). Therefore, critical mental health education is vital to be considered because when the students are mentally fit, they will not be involved in any activity related to politics in which they believe they have nothing to gain (12, 21, 52). In this way, the students' self-motivation and awareness sense must be provided to improve their performance by not getting involved in any political activity (12, 60). The ideological passion is critical to understand for the students related to politics. However, they must be limited in their learning and should not get involved in utilizing the internet for political purposes.

In the same way, for the wellbeing of the students, the appropriate resources must be utilized effectively and provided with a reliable opportunity for the students to enhance their critical thinking ability by reducing their political influence (75–77). Significantly, the responsibility of the management is to design the curriculum in a way that must let the students seek insights from all nukes and corners of the social fabric for improved academic performance. Also, the controlled use of social media platforms must be ensured to let students keep their academic performance and psychological states in check. Interestingly, by developing such strategies, the students can improve their performance in a productive way for the more significant benefit of society (8, 10–12, 28).

***H2*.*** There is a moderating role of ideological passion in the relationship between mental health education and college students' wellbeing*.

***H3*.*** Politically motivated internet addiction is moderating in related mental health education and college students' wellbeing*.

## Methodology

### Prepare questionnaire

In this study, the questionnaire was prepared for the five-point Likert scale. This type of questionnaire is considered appropriate to collect data from a large population in a reliable way. Indeed, the survey-based questionnaire is essential to collect the data from the respondents because it is easy to distribute the questionnaire and collect it back for the theater analysis (78, 79). In this way, the current study adopted the same data collection method. The scale items were taken from different studies with careful consideration and face validity to effectively collect the data from the respondents. The scale items for mental health education were adapted from the study (80). Further, the scale items for students' wellbeing were adopted from the study (81). In the same way, the scale items for politically motivated internet addiction were adopted from the study (82). Lastly, the scale items for ideological passion were adopted from the study (83). In this way, the scale item for mental health education was taken to determine the relationship between mental health education and the wellbeing of the students.

Similarly, the scale items for the wellbeing of the students were carefully taken to understand the critical role of students' wellbeing and the student's motivation. This study carefully adopted scale items to measure the relationship effectively. In addition, the scale items for ideological passion were adopted to measure the moderating role of ideological passion in the relationship between mental health education and the wellbeing of students. Lastly, the scale items for politically motivated internet addiction were adopted to collect the data to understand its moderating role in the relationship between mental health education and the wellbeing of students. Furthermore, to test the face validity of these scale items, the experts' opinion was considered, and the research experts were contacted to provide their opinions on the face validity of the questionnaire. In this way, by getting positive responses from different experts, these scale items were incorporated into the questionnaire to effectively collect the data from the target respondents.

### Data collection process

In this section of the study, the detail of the data collection process is presented. Firstly, the current study respondents were students of different colleges in China. Therefore, the random sampling technique was adopted, and students were given the questionnaire randomly (84). Questionnaires were distributed to them, and they were provided with a brief introduction to the study to gain familiarity with it. Also, the individuals were allowed to ask any question related to the study, with difficulty responding to the questionnaire. In this way, the expected response rate for mental health studies was 50% in light of the earlier studies. In this regard, 750 questionnaires were provided to the students with the technique of random sampling, as it is appropriate to collect the data from a large population. Similarly, 330 questionnaires were taken back from the students, and 320 responses were analyzed for this study. Finally, the students were thanked for their precious time and contributed to the study by the researcher.

## Findings

### Convergent validity

**Figure 2:**
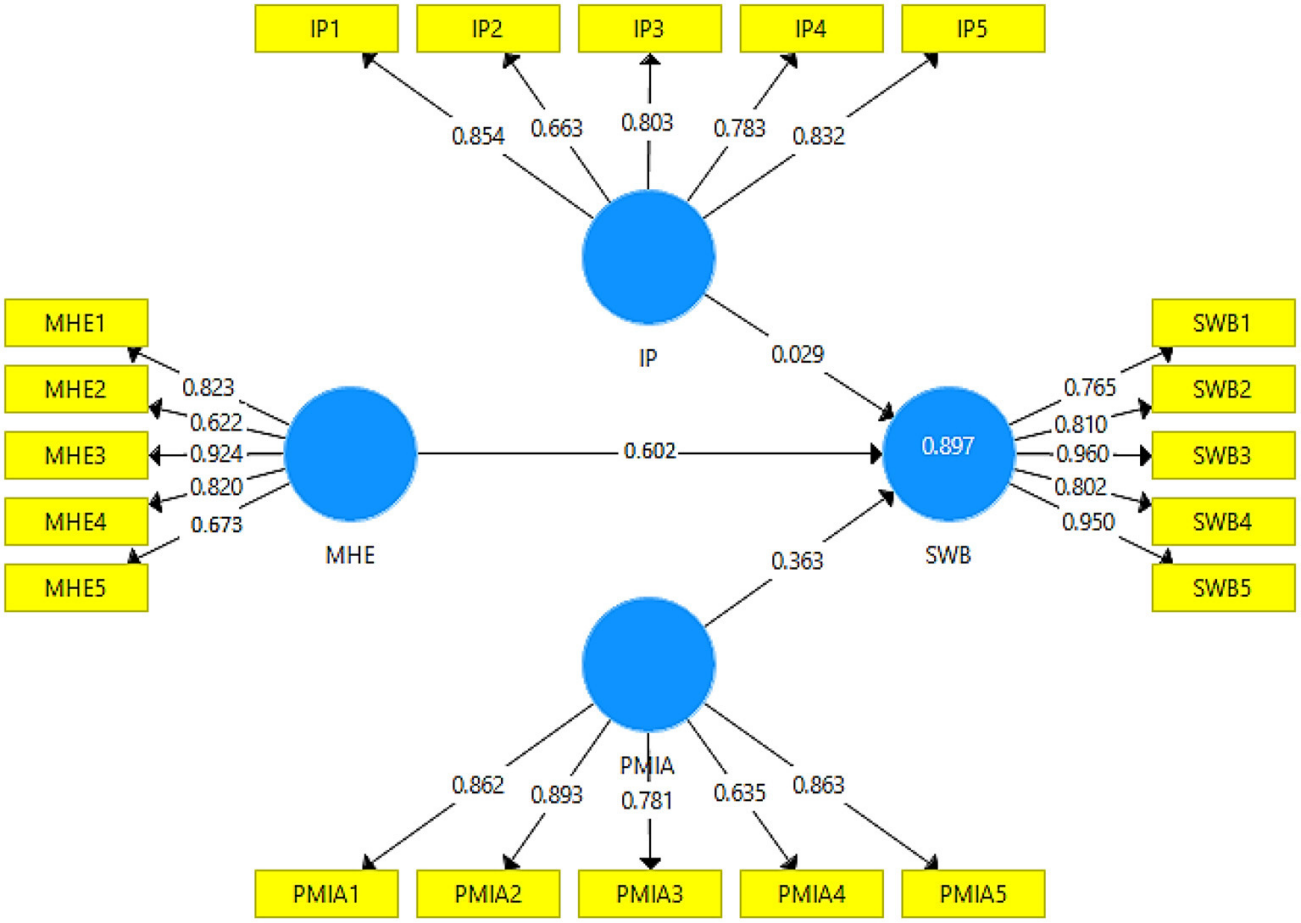
Measurement model. MHE, Mental Health Education; IP, Ideological Passion; SWB, College Student's Wellbeing; PMIA, Politically Motivated Internet Addiction.

This section explains the convergent validity results to check the reliability and validity of the scale items used for each variable to collect the data for the questionnaire. In this way, PLS algorithm calculations were considered to check the factor loadings, composite reliability (CR), and average variance extracted (AVE) available in Figure 2. In addition, the values of factor loadings for each scale item were not <0.60 recommended by Yingfei et al. (85). Similarly, the values of CR were more significant than 0.70 for each variable used in the study, as recommended by Henseler and Fassott (86). Also, the AVE value for each scale item was more significant than the 0.50 recommended by Ramayah et al. (87) for advanced studies. Therefore, the results demonstrate evident reliability and validity (see Table 1).

**Table 1:** Constructs, factor loadings, CR, and AVE.

**Variables**	**Items**		**Factor loading**	**Alpha**	**CR**	**AVE**
Ideological passion	IP1	I believe that teachers exist to serve their students.	0.854	0.859	0.892	0.624
	IP2	I have a strong urge to become a good person.	0.663			
	IP3	I am engrossed with the idea of people listening to me when I talk.	0.803			
	IP4	I am too exacting in my school work.	0.783			
	IP5	I get upset when I miss a class.	0.832			
Mental health education	MHE1	I would go for professional help in the case of a serious emotional problem.	0.823	0.813	0.872	0.584
	MHE2	I would feel comfortable talking about personal problems with a professional.	0.622			
	MHE3	There are mental health services available in my community.	0.924			
	MHE4	Mentally ill people are crazy.	0.820			
	MHE5	There are sufficient existing services for the mentally ill.	0.673			
Politically motivated internet addiction	PMIA1	I spend more time on internet than I previously planned.	0.862	0.868	0.905	0.660
	PMIA2	My tasks may be interrupted due to I spend too much time on the internet.	0.893			
	PMIA3	The people in my life complain about too much time I spend on the internet.	0.781			
	PMIA4	My professional success and productivity are negatively affected by the internet.	0.635			
	PMIA5	I wanted to reduce the time I spent on the internet and I failed.	0.863			
College student's wellbeing	SWB1	I don't have problems to meet the standards in school.	0.765	0.911	0.934	0.742
	SWB2	I can solve the learning problems easily.	0.810			
	SWB3	I am able to achieve as good as most of my class mates.	0.960			
	SWB4	I am satisfied about the development of my academic goals.	0.802			
	SWB5	I am optimistic about the next school years/about the time after school.	0.950			

MHE, Mental Health Education; IP, Ideological Passion; SWB, College Student's Wellbeing; PMIA, Politically Motivated Internet Addiction.

### Discriminant validity

This section of the study provides the results of discriminant validity that were checked to determine the distinction for the scale items used (see Table 2). In this way, the more advanced and realistic approach Heteritrait-Monotrait (HTMT), was used to check the discriminant validity for the scale items. Therefore, results show that the values of discriminant validity for each scale item were <0.90, which is recommended by Hair et al. (88).

**Table 2:** Discriminant validity.

	**IP**	**MHE**	**PMIA**	**SWB**
IP				
MHE	0.728			
PMIA	0.821	0.816		
SWB	0.768	0.762	0.712	

MHE, Mental Health Education; IP, Ideological Passion; SWB, College Student's Wellbeing; PMIA, Politically Motivated Internet Addiction.

### The PLS-SEM results

**Figure 3:**
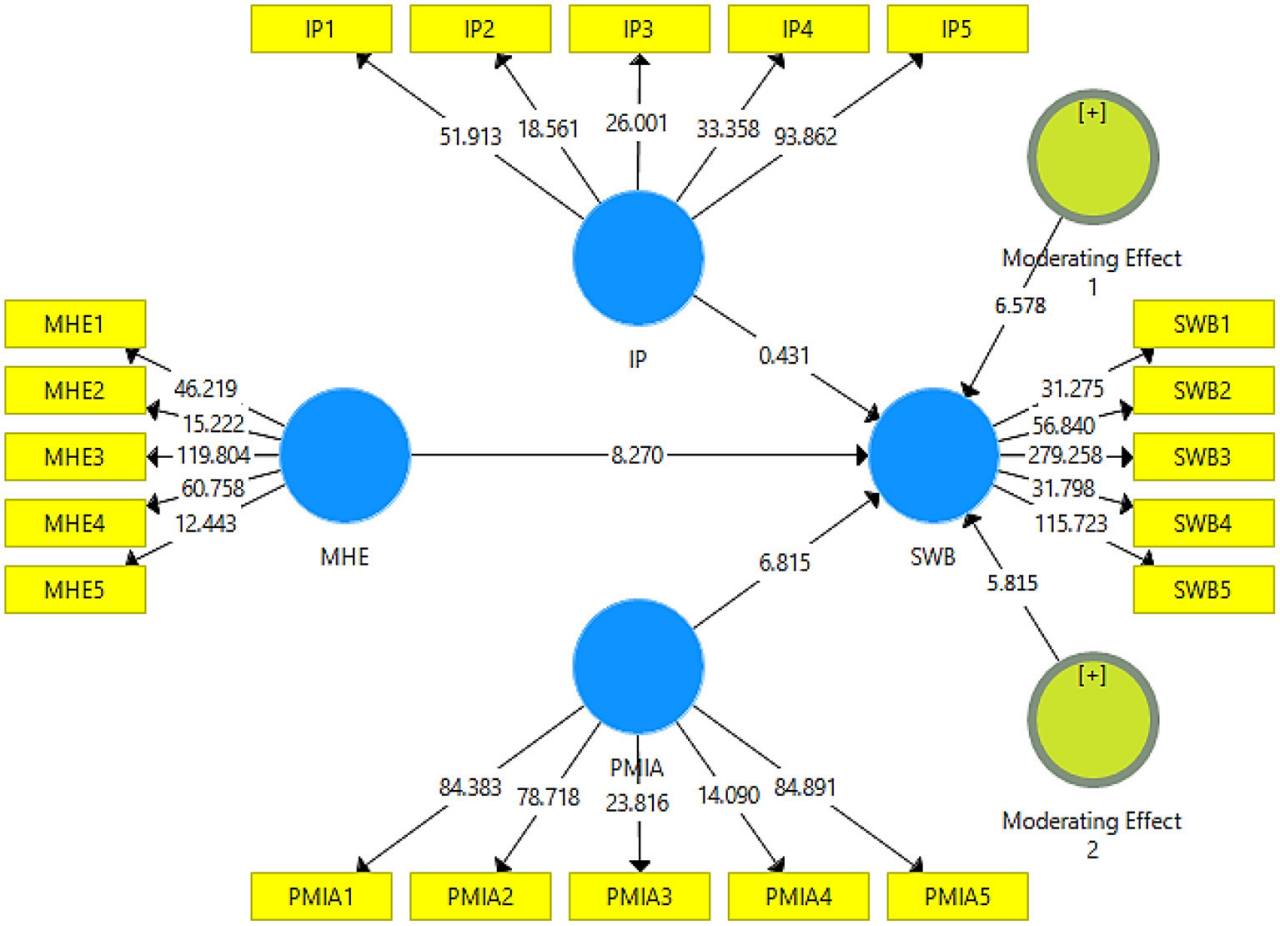
Structural model. MHE, Mental Health Education; IP, Ideological Passion; SWB, College Student's Wellbeing; PMIA, Politically Motivated Internet Addiction.

**Figure 4:**
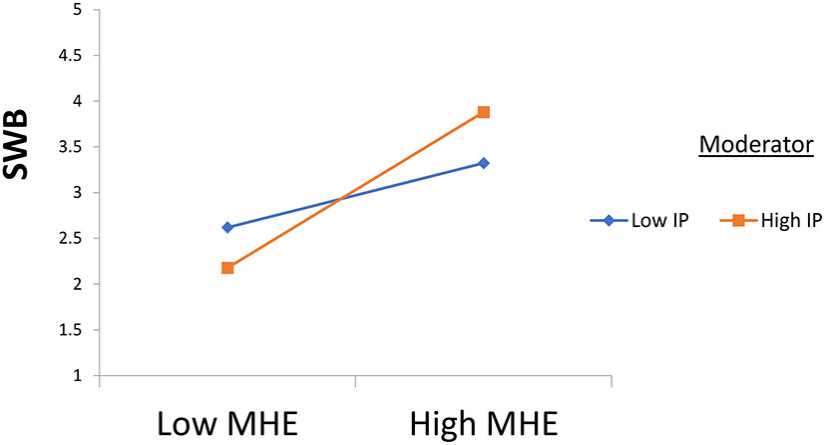
Moderation 1. MHE, Mental Health Education; IP, Ideological Passion; SWB, College Student's Wellbeing; PMIA, Politically Motivated Internet Addiction.

**Figure 5:**
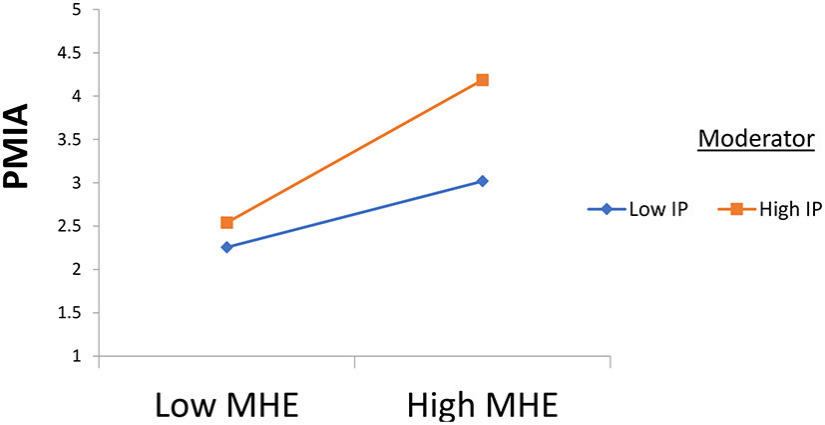
Moderation 2. MHE, Mental Health Education; IP, Ideological Passion; SWB, College Student's Wellbeing; PMIA, Politically Motivated Internet Addiction.

This study used partial least squares (PLS) to analyze the data (see Figure 3). At first, hypothesis 1 was tested, and the results revealed a significant relationship between mental health education and students' wellbeing (β = 0.602, *t* = 8.270, and *p* = 0.000). Also, hypothesis 2 was tested, and the results reveal the significant moderating role of ideological passion in the relationship between mental health learning and students' wellbeing (β = 0.250, *t* = 6.578, and *p* = 0.000). In the end, hypothesis 3 was tested, and the results reveal the significant moderating role of politically motivated internet addiction in the relationship between mental health education and students' wellbeing (β = 0.221, *t* = 5.815, and *p* = 0.000) results available in Table 3. On the one hand, the results reveal that ideological passion positively strengthens the relationship between mental health education and students' wellbeing (see Figure 4). On the other hand, the results demonstrate that politically motivated internet addiction positively strengthens the relationship between mental health education and students' wellbeing (see Figure 5).

**Table 3:** Hypotheses test.

**Hypotheses**	**Beta value**	**(STDEV)**	***T*-values**	***P*-values**	**Status**
**H1**. MHE -> SWB	0.602	0.073	8.270	0.000	Significant
**H2**. Moderating Effect 1 -> SWB	0.250	0.038	6.578	0.000	Significant
**H3**. Moderating Effect 2 -> SWB	0.221	0.038	5.815	0.000	Significant

MHE, Mental Health Education; IP, Ideological Passion; SWB, College Student's Wellbeing; PMIA, Politically Motivated Internet Addiction.

## Discussion

The results of H1 supported a relationship between mental health education and college students' wellbeing. It is critical to understand that the development of the mental ability of the students is based on the mental education they are provided by the educational institutes, as discussed in the study (9). The students working for their mental wellbeing to improve their performance in the classroom activities are highly passionate about effectively utilizing the internet resources to improve their performance for their better output highlighted by Dias et al. (10). On the other hand, the students willing to be involved in politics are not focusing on their career and physical development (13). In this regard, the mental condition of the students is failed as they are not provided with the appropriate resources to improve their productivity. The students in Denmark and Norway are provided with the opportunity to utilize the internet and get related information about mental health literacy because it is believed that with the help of mental health literacy, the students can improve their performance, as discussed by the study of Rudaz et al. (13). Oppositely, the students with very little good mental health are not self-motivated to improve their wellbeing because they are not provided with the opportunity to improve their capability effectively (68). However, government policies are critical to ensure students work the right way and are passionate about politics. However, how they are involved in politics is not influencing their mental health (54). As a result, the more mentally strong students perform, the better their learning, ultimately leading them to successful careers. Interestingly, it is the responsibility of the parents to motivate the students for their product development and utilize digital tools for their better learning and advanced training.

The results of H2 highlight a moderating role of ideological passion in the relationship between mental health education and college students' wellbeing. Similarly, the results of H3 highlight a moderating role of politically motivated internet addiction in related mental health education and college students' wellbeing. It is a fact that in our society, the students are more influenced by the ideological passions of their favorite personalities because they consider them the hero, and they want to follow in their footsteps to get success (9, 66). It is the basic human instinct that people are motivated when they have interacted with the people of their community and have the same mental understanding. In the same way, the students are motivated by the political leaders, influencing the other people to get the same affiliation with those particular leaders, as discussed by the study (52). However, it is the academic institute, parents, and government's responsibility to be mindful that students should only be using such political passions for positive academic reasons (10). The political leaders develop different perceptions according to their minds and the party's policy. However, the students are influenced and do not think critically and realistically analyze the thing. However, political leaders are highly involved in propaganda, so students need to be vigilant about the difference between a positive and not-so-positive political agenda (66). It is critical to understand that the students must be enlightened to be associated only with those political passions which are helpful for their academic endeavors and will help them perform better through strengthened mental health (9, 54, 66).

Indeed, such passion of the students is realistic as the students of America are primarily involved in political activities, separating the critical success factor and the internet for the study (89). In the same way, students of Asian countries, particularly those of India and Nepal, are also involved in political activities, assisting in their mental health management (10, 13, 52). However, parents of students concerned must focus on their mental health development by providing them with mental health literacy (90). When the students should be provided with such opportunities to increase their mental health, with the help of effective management and mental health literacy, they will be able to think critically and realistically analyze the thing.

Moreover, the government is responsible for establishing policies for the students to improve their mental capability and control political affiliations (9, 52). As a result, students would be highly involved in their study material and utilize the internet by not being involved in undue political activities. Likewise, in Russia, students are not actively allowed to participate in unchecked and unmonitored political activities because such political associations can conversely negatively affect students' mental health and academic performance (10, 53). Notably, the teacher's responsibility also lies in that they must help students find mentors and leaders from the political exchequer of the country from whom they can seek positive passion and mentorship. In this regard, a reward system must be introduced for the students to participate actively in class activities (17, 27, 91, 92). In this way, the student would develop their mental health for better learning and better wellbeing in the long term.

## Conclusion

This study identified a critical role of students' mental health in the wellbeing of the students for their learning and improving their skills. However, it is also a fact that politically motivated internet addiction and ideological passion are essential in the relationship between students' mental health education and their wellbeing. Indeed, the students involved in controlled political affiliations have better critical thinking abilities and mental strength for increased academic performance. However, the critical role of internet addiction is crucial if it is positive and is in line with students' mental health requirements. In this regard, if the political leaders motivate the students to utilize the internet for the benefit related to the course material, the student would not excessively get an addiction to the internet. However, they would limit it to getting mental health education and health literacy to anticipate and solve mental health problems. Understanding that the students are highly motivated by different leaders because they have ideological passions is critical. Though, if their passion is turned into their learning, the student's performance would be increased in an effective way that would be appropriate for their development of skills and wellbeing. In addition, the study highlights that the students must be motivated by their parents and their family to effectively develop their critical thinking ability and mental health assessment to participate in class activities and improve their learning standards.

In this way, not only would the student's performance be increased, but they would be able to participate in the extra-curricular activities related to the study material to improve their skills to the advanced level for competing with the international students and getting a satisfactory office. Moreover, it is the government's responsibility to ensure that the students are provided with adequate mental health education and training sessions because the thin sections of mental health are essential for the fundamental development of the students. The students with high mental health ability are more self-motivated and participate effectively in different activities to improve their performance and contribute to the benefit of the people.

## Implications

### Theoretical implications

This study has significant theoretical implications related to the mental health development of the students and the wellbeing of the students in the context of China. However, these implications can be generalized and applicable to knowledge worldwide. It is critical to understand that this study provides a robust theoretical framework for the knowledge and the literature related to the relationship between mental health development and the wellbeing of Chinese students. Furthermore, this study contributes to the literature by providing the significant moderating role of politically motivated internet addiction in the relationship between mental health education and the wellbeing of Chinese students. In this regard, in the previous literature, up to the researcher's knowledge, very few studies were conducted to determine the relationship between mental health education and the students' wellbeing in China. At the same time, literature is scarce on the use of social media and its role in shaping students' mental health through mental health education. Remarkably, this study provides an appropriate relationship between the different variables taken in the study's theoretical framework in a single dimension to enhance knowledge and contribute to the literature for future studies and researchers.

Moreover, the scope of this study is limited to improving the mental health education and the wellbeing of the Chinese students effectively for improving the capability and skills to improve their performance constructively. At the same time, this study highlights the significant moderating role of ideological passion in the relationship between mental health education and the wellbeing of Chinese students that earlier studies did not address areas up to the researcher's knowledge. This study contributes to the literature to improve the theory of the wellbeing of students with the help of internet addiction, which is politically motivated. This study is significant as it provides an important theoretical implication that is realistic and important for the literature and knowledge.

### Practical implications

The current study is significant because it provides the crucial practical implication that stakeholders consider proving the productivity and wellbeing of Chinese students learning in different colleges and schools. This study highlighted that the positive use of the internet could enhance the students' experience related to their security development and better performance in classroom activities. Secondly, this study highlights that it is the responsibility of the parents to motivate students to seek inspiration from academic and social-political mentorship. Similarly, it is also the responsibility of the teachers to provide adequate guidelines to the students to utilize the internet meaningfully and effectively. By taking help from these resources, the students must improve their performance productively. Furthermore, students exposed to efficient social media tools and external mentorship perform comparatively better than those who do not seek assistance from internet usage and ideological passion.

Additionally, this study demonstrates that it is also the government's responsibility to ensure that the schools and colleges are providing mental health education to the students. Because with the help of mental health education, the students can learn about their mental condition and utilize it in the best way to improve their performance and wellbeing. Indeed, it is also the students' responsibility to get involved in mental health education and use the internet positively to learn the course material and improve their skills for better performance and a better future. Significantly, these practical implications of the study are not limited to China. Nevertheless, the students of other countries can also improve their mental health for their wellbeing through practical social media usage and required ideological passion.

## Limitations and future directions

This study highlighted the relationship between mental health education and the moderating role of ideological passion and politically motivated internet addiction. On the other hand, during the literature review and the discussion of this study, it was identified that significant other variables are influencing college students' wellbeing. In this way, future studies must consider the moderating role of the learning environment in the relationship between mental health education and college students' wellbeing. Similarly, the studies need to focus on the role of extrinsic motivation as a moderator in the relationship between mental health education and college students' wellbeing. Additionally, the researchers need to focus on the mediating role of student cognitive ability in the relationship between mental health education and college students' wellbeing. In this way, it would help contribute to the body of knowledge on the phenomena at hand.

## Data availability statement

The original contributions presented in the study are included in the article/supplementary material, further inquiries can be directed to the corresponding author.

## Ethics statement

The studies involving human participants were reviewed and approved by Xinyang Normal University, China. The patients/participants provided their written informed consent to participate in this study. The study was conducted following the Declaration of Helsinki.

## Author contributions

SM: conceptualization, data collection, and draft writing. The author approved the final version for publication.

## Conflict of interest

The author declares that the research was conducted in the absence of any commercial or financial relationships that could be construed as a potential conflict of interest.

## Publisher's note

All claims expressed in this article are solely those of the authors and do not necessarily represent those of their affiliated organizations, or those of the publisher, the editors and the reviewers. Any product that may be evaluated in this article, or claim that may be made by its manufacturer, is not guaranteed or endorsed by the publisher.
